# Towards T7 RNA polymerase (T7RNAP)-based expression system in yeast: challenges and opportunities

**DOI:** 10.1080/21655979.2023.2180579

**Published:** 2023-04-27

**Authors:** David Sáez Moreno, Udi Qimron, Joana Azeredo, Lucília Domingues

**Affiliations:** a CEB—Centre of Biological Engineering, University of Minho, 4710-057, Braga, Portugal; b LABBELS—Associate Laboratory, 4835-198, Guimarães, Braga, Portugal; c Department of Clinical Microbiology and Immunology, Sackler School of Medicine, Tel Aviv University, Tel Aviv, Israel

**Keywords:** Synthetic biology, yeast engineering, T7 RNA polymerase, protein expression, mRNA capping

## Abstract

During the last decades, we have witnessed unprecedented advances in biological engineering and synthetic biology. These disciplines aim to take advantage of gene pathway regulation and gene expression in different organisms, to enable cells to perform desired functions. Yeast has been widely utilized as a model for the study of eukaryotic protein expression while bacteriophage T7RNAP and its promoter constitute the preferred system for prokaryotic protein expression (such as pET-based expression systems). The ability to integrate a T7RNAP-based expression system in yeast could allow for a better understanding of gene regulation in eukaryotic cells, and potentially increase the efficiency and processivity of yeast as an expression system. However, the attempts for the creation of such a system have been unsuccessful to date. This review aims to: (i) summarize the efforts that, for many years, have been devoted to the creation of a T7RNAP-based yeast expression system and ii) provide an overview of the latest advances in knowledge of eukaryotic transcription and translation that could lead to the construction of a successful T7RNAP expression system in yeast. The completion of this new expression system would allow to further expand the toolkit of yeast in synthetic biology and ultimately contribute to boost yeast usage as a key cell factory in sustainable biorefinery and circular economy.

## GRAPHICAL ABSTRACT

**Figure:**
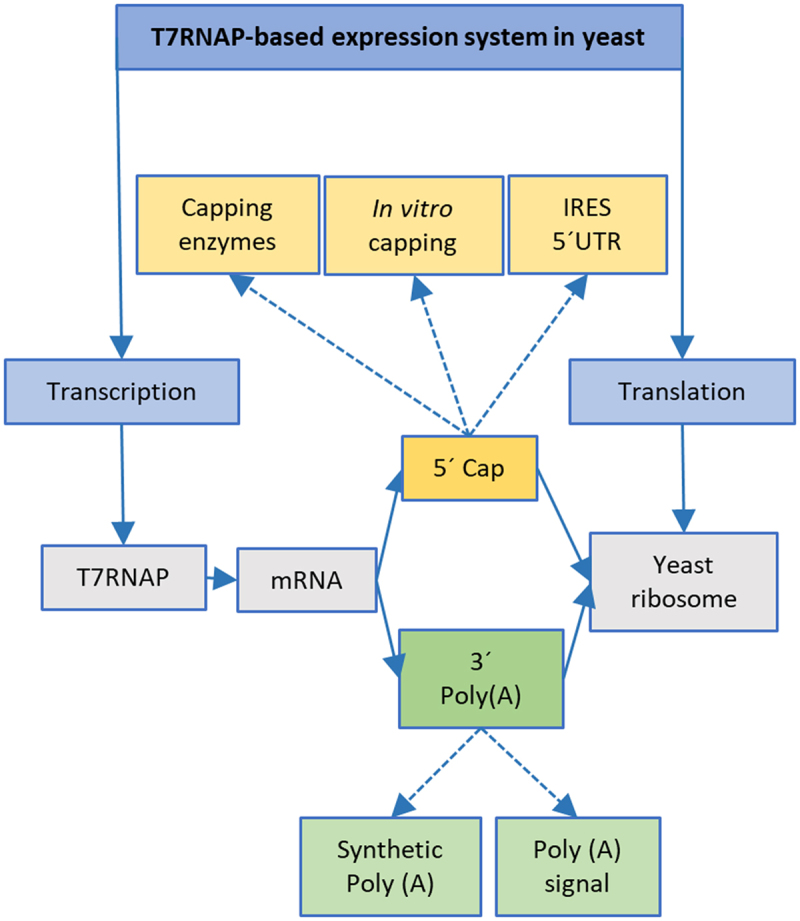

## Highligths

1. A T7RNAP-based protein expression system has never been effectively developed in yeast despite several attempts.
2. A possible strategy to achieve cap-dependent translation in yeast could be to fuse T7RNAP to capping enzymes.
3. Capping enzymes can be tethered to RNA binding domains designed in the target transcript generated by T7RNAP in yeast.
4. To achieve cap-independent translation, IRES might be used in the 5’ UTR upstream of the target gene.

## 1. Introduction

An orthogonal genetic system is a genetically engineered system composed of independent biological elements. Such genetic systems aim to complete biological functions in a host (replication, transcription, and translation), without compromising, nor being compromised, by those of the host cell [1]. This would allow for better control of over-expressed circuits and the expansion of novel cellular functions [2].

In the development of orthogonal gene expression systems, bacteriophage T7 RNA polymerase (T7RNAP) is the basis and the preferred system for the transcription process. It has been extensively studied and its wide use relies mainly on (i) the specificity toward a relatively small promoter [3,4], (ii) its high processivity [5], (iii) its single subunit nature, like T3, K11, and SP6 phage polymerases [6], (iv) the fact that it does not need initiation proteins [7] and (v) the divisibility of its structure [8].

An example of its use is the pET-based expression systems in bacteria, where the T7RNAP is inserted in the chromosome of the bacterium *Escherichia coli*, and it recognizes and transcribes the gene of interest downstream of the T7 promoter, present in a plasmid [9]. The development of this expression system has been pivotal for modern biotechnology. T7 RNAP has been shown to be compatible with many other prokaryotic [10,11] and eukaryotic hosts [12]; however, the post-transcriptional processing of mRNA in eukaryotes has been a burden on the development of T7RNAP-eukaryotic expression systems. For further reading on orthogonal uses of T7RNAP in different hosts, we recommend the following reviews [13,14].

Yeast, unicellular eukaryotic microbe, is a well-studied model organism. Its versatility has allowed yeast to play a role in the development of many tools and concepts in synthetic biology [15,16] and is widely utilized as a protein expression system. Although the choice of host for protein production depends on the target protein, yeast offers several advantages that make it an attractive host [17]. Yeast can carry on post-translational modifications and grows robustly in a relatively inexpensive medium [18,19]. *Saccharomyces cerevisiae* and *Pichia pastoris* are the yeasts most used as hosts for protein production [17]. They have generally recognized as safe (GRAS) status and have become simple to genetically manipulate [2,18,20]. Moreover, they play an important role in modern biotechnology and in sustainable processes like biorefinery, bioethanol production, and bioremediation [21–23]. In the quest for sustainable development, the yeast *S. cerevisiae* has emerged as a robust cell factory that can drive the biotechnological production of biofuels [21,24] and chemicals [25] from renewable substrates such as industrial wastes and lignocellulosic biomass. The growing portfolio of novel enzymes catalyzing previously unknown reactions, engineered enzymes with enhanced performance and novel synthetic pathways designed for overproduction of biofuels and/or chemicals will enable its sustainable production [26] if efficient expression systems are available. High yields and low cost are a requirement for protein expression and the biomanufacturing industry. The development of a new protein expression system based on T7RNAP and yeast could meet this demand.

A fully functional T7RNAP-based expression system in yeast could therefore bring many benefits in different fields. First, the specificity of the T7RNAP/T7 promoter could provide better control and regulation of the expression [27]. Second, the efficiency of yeast as a protein expression system could be potentially increased, since the T7RNAP/T7 promoter pair has previously been shown to be more processive than endogenous promoters for RNA polymerase I in other lower eukaryotes [28]. Third, the impact of overexpression on the host could be reduced since the orthogonalization of transcription avoids the use of the native RNA Polymerase II, since the orthogonalization of transcription avoids the use of the native RNA Polymerase II (RNA pol II) [2]. Third, it could allow for a better understanding of gene regulation in eukaryotic cells [29] and increase the toolbox of yeast for synthetic biology. Lastly, this development could help increase the versatility and efficiency of yeast, potentially improving their role in the sustainable production of metabolites [21,26].

Orthogonal systems are foundational for synthetic biology and T7RNAP has paved the way for the construction of new systems [30]. One example is the transcription-translation network developed by An and Chin [31] in which protein expression relies on AND logic gates, where the target protein is only translated if T7RNAP and an O-ribosome are present. Shis and Bennett [32] also used AND logic gates to regulate transcription of a split T7RNAP.

An orthogonal T7RNAP transcription system in yeast has been utilized by many researchers (Table 1), and it has been shown that the enzyme is able to integrate into the yeast chromosome. Once it is expressed, it can reach the nucleus with a nuclear location sequence (NLS) and recognize its promoter, generating transcripts. However, the T7RNAP-transcribed mRNA does not get translated into proteins [35,36], thus resulting in a nonfunctional expression system. T7RNAP evolved in prokaryotes and this represents the main challenge to render the system functional. The transcripts should be made suitable for recognition of the eukaryotic translation machinery, by proper capping and polyadenylation.

**Table 1.** Summary of relevant findings from the use of T7RNAP orthogonal transcription system in yeast.

Main findings	T7RNAP-based transcription	T7RNAP-based protein expression	Host	Reference
-T7 RNA polymerase produced in yeast can enter the nucleus without a NLS and can initiate transcription from its own promoter. - The level of target RNA is proportional to the amount of T7 RNA polymerase that is produced.	Achieved	Not achieved	*S.cerevisiae*	[33]
- T7RNAP remains mostly cytoplasmic when NLS is not present. - T7RNAP without NLS are produced in greater quantity, do not seem to produce stress responses, and seem to enter the nucleus passively if enough quantity is produced. - mRNA accumulates in cells but is not translated. However, target transcripts were produced in more quantity for NLS containing T7RNAP, when target DNA is integrated into the chromosome. - When T7RNAP was expressed in a plasmid, the target mRNA was created at similar levels with or without the NLS, indicating passive diffusion. - T7 terminator was correctly recognized.	Achieved	Not achieved	*S. cerevisiae*	[34]
- Transcripts of T7RNAP are not capped. However, they can undergo nuclear export (Rip1p based export), splicing and poly(A), providing the right eukaryotic signals. - Proper 3`end formation (poly(A)) is enough for export, since putting a terminator before the poly(A) signal made all transcripts nuclear. It does not require RNA pol II. - Terminating the T7 transcription with a T7 terminator, make the transcripts accumulate in the nucleus, due to the lack of poly (A) formation.	Achieved	Not achieved	*S. cerevisiae*	[36]
-T7RNAP can be expressed in *P. pastoris* and generate mRNAs. - To drive translation in the absence of cap, the authors added EMCV or HCV IRES upstream of the reporter gene. - By adding a control of non-T7RNAP, they found that the gene is also being expressed, indicating a T7-independent translation. - Used RACE to detect transcription start sites, and found that the IRES had promoter activity, and was recruiting RNA Pol II, thus driving translation.	Achieved	Not achieved	*P. pastoris (Komagataella phaffii)*	[35]
- HIV viroporin can permeabilize the nuclear membrane to achieve higher mRNA export.	Achieved	Not demonstrated	*S. cerevisiae*	[37]

The aim of this review is (i) to contextualize and evaluate the efforts devoted to the creation of a T7RNAP-based yeast expression system and the learnings thereof and (ii) to review the latest advances in yeast transcription and translation knowledge for suggesting future directions for the development of a successful T7RNAP-based expression system.

## 2. Cap-dependent translation

The 5´cap, in eukaryotes, is the first post-transcriptional modification of the nascent mRNA. It plays essential roles in mRNA transport to the cytoplasm, processing, translation efficiency, and protection from 5´exonucleases degradation [38]. The capping process is conserved in eukaryotes and is carried out by three enzymes, whose structure and genetic organization differ between species [38]. Yeast encode a three-component capping system: first, an RNA Triphosphatase (TPase) that hydrolyses the 5´end of the nascent mRNA, releasing inorganic phosphate and a di-phosphate terminated transcript. Second, an RNA guanylyltransferase (GTase) that caps the transcript with guanosine monophosphate (GMP). Lastly, a methyltransferase (MTase) that methylates the terminal Guanine base in the N7 position. The cap formed is referred to as Cap 0 or m^7^GpppN and is the main cap present in lower eukaryotes [39].

In *S. cerevisiae*, specifically, the enzyme Cet1 acts as the TPase, Ceg1 as GTase and Abd1 as MTase. The capping enzymes are recruited by the transcription complex; contrary to what happens in higher eukaryotes, it is the GTase Cet1 that binds directly to the C-terminal domain (CTD) of RNA pol II, provided that the Ser5 of the CTD is phosphorylated [40,41]. Cet1 catalyzes the TPase reaction, hydrolyzing the 5´triphosphate end of the transcript into a 5´di-phosphate end. Cet1 allosterically activates Ceg1. Then, Cet1 uses GMP to cap the 5´end of the transcript [42]. Finally, the MTase, Abd1, which also interacts with the phosphorylated CTD of RNA pol II, transfers a methyl group from S-adenosylmethionine to the guanine base in the position N7 [43,44]. In yeast, this process forms a mature RNA with cap 0 mRNA or m^7^G cap.

For the cap-dependent translation initiation, the ribosome is recruited to the mRNA by the yeast eukaryotic initiation factor (eIF4) group. The eIF4F polypeptides eIF4A, eIF4E, and eIF4G mediate translation initiation by recruiting the 40S ribosomal subunit to the 5´end of the mRNA [45]. Poly(A)-binding protein (Pab1) promotes the assembly of ribosomal subunits onto the mRNAs, through what is termed ‘the closed-loop model,’ where eIF4G interacts with the 5´cap of the mRNA and eIF4E interacts with the 3´-poly(A) and a circular mRNA structure is formed. This facilitates the recruitment of ribosomal subunits, leading to protein synthesis initiation [46].

## 3. Cap-independent translation

In eukaryotic mRNA, immediately upstream of open reading frames, there are sequences that do not code for proteins. Instead, they play an important role in regulation and especially in translation initiation. They are called leader sequences or 5´UnTranslated Regions (5´UTRs). In addition to their role in the regular cap-dependent mRNA translation, some of them can drive cap-independent translation [47].

Some eukaryotic viruses, despite not having their own mRNA capping system, are able to translate proteins and eventually replicate in the cytoplasm [48,49]. The motifs that allow them to overcome cap deficiency are the Internal Ribosome Entry Sites (IRES) in their 5´UTRs. IRES do not rely on the cap for translation, in contrast, they form complex RNA structures that interact with the ribosomes, other proteins or other RNAs. IRES can promote internal (cap-independent) translation initiation in two different manners depending on its type, by directly interacting with the 40S ribosomal subunit, or by binding to eIFs and RBPs, which then recruit the 40S subunit [50]. This contrasts with the cap-dependent translation, in which the 40S ribosomal subunit recognizes the cap and binds to the 5´UTR, then starts the scanning for the start codon. Only when it reaches it, the elongation starts by the formation of the 80S ribosome (see the topic reviewed) [49].

In the context of synthetic biology, IRES are often included in eukaryotes between open reading frames (ORFs) to allow expression of several proteins in polycistronic mRNA. The first ORF is translated by cap-dependent mechanisms and IRES drive the translation of the other ORFs through a cap-independent mechanism [51,52].

Scanning mechanisms of translation initiation are still an elusive process. Another mechanism of IRES-independent and cap-independent translation in eukaryotes is driven by cap-independent translational enhancers (CITEs). CITEs are found in the 3´UTRs of plant viral RNAs and they drive translation by recruiting the translational apparatus and driving it to the 5´end of the mRNA, by base–pair interactions. For more details, refer to [49,53,54].

## 4. The state of the art of orthogonal T7-RNAP transcription in yeast

Back in 1987, Chen et al. demonstrated that T7RNAP could be expressed in the yeast *S. cerevisiae*, where it could initiate transcription from a T7 promoter in the yeast nucleus. However, the mRNA produced from the transcription of T7RNAP was not translated into protein. Since then, a handful of researchers have tried to improve this system to make T7RNAP more efficient in yeast without success (Table 1). To the best of our knowledge, there is no report to date demonstrating the construction of a T7RNAP-based protein expression system in yeast.

The first reported study [33] showed that T7RNAP could access yeast chromosomal DNA and that its transcription could be stimulated by providing better access to chromatin. In fact, T7RNAP transcription is less effective in the nuclear chromatin of higher eukaryotes, contrary to lower eukaryotes [28,55,56].

Benton *et al*. [34] improved this system by introducing a nuclear location signal (NLS) to direct the phage polymerase to the yeast nucleus, resulting in 5–10-fold the mRNA quantity produced from a chromosomal target gene. They also introduced a T7 terminator in the construction of their target DNA, which was correctly recognized by T7RNAP, reducing the size of the long transcripts that were being produced. However, also those transcripts were not translated into protein.

In an extensive study, Dower & Rosbash [36], identified two key points for the failed T7RNAP-transcript translation, (i) the export of mRNA from the nucleus to the cytoplasm and (ii) the capping of the transcript for ribosomal recognition. They showed that transcripts were polyadenylated if they added a eukaryotic terminator (with its polyadenylation signal) and this promoted their export to the cytoplasm. The transcripts remained nuclear when the T7 terminator, lacking the polyadenylation signal was used, highlighting the need for polyadenylation for nuclear export. To overcome the need for capping the T7RNAP transcripts, they designed a stem-loop in the target RNA and co-expressed the corresponding RNA binding protein fused to a Cbp20p (cap-binding protein). These efforts, however, did not lead to a successfull protein translation. However, they strongly suggest the need for both capping and polyadenylation of the mRNA to achieve translation from the T7RNAP-based system.

In a more recent study, Hobl *et al*. [35] aimed to establish the first T7RNAP-based expression system in *P. pastoris*. They showed the nuclear location of T7RNAP in *P. pastoris* and the ability of the enzyme to recognize its promoter and generate mRNAs. To tackle first the nuclear export of the transcripts, they inserted the AOX1 terminator sequence downstream of the reporter gene, carrying a polyadenylation signal. Second, to work around the need for capping the transcripts, they aimed to drive translation in the absence of a cap by using a viral IRES upstream of the reporter gene. This led to protein expression apparently driven by T7RNAP. However, the expression is not T7RNAP-dependent; the negative control, a yeast not expressing T7RNAP but carrying the reporter gene construction also showed transcript expression. Follow-up Rapid Amplification of cDNA Ends (RACE) experiments revealed that the IRES used upstream of the reporter gene, from the encephalomyocarditis virus (EMCV) and hepatitis C virus (HCV) had promoter activity, thus recruiting RNA pol II and driving T7-independent expression.

Finally, the last report to date involving T7RNAP transcription in yeast [37], aimed to construct a stable expression system. In the first instance, their T7RNAP-based system indicates successful protein translation. However, to achieve cap-independent translation, they also used viral IRES. A critical analysis of the study reveals that the IRES sequence used upstream of their target gene (EMCV IRES) was the one previously identified by Hobl *et al*. [35] to have promoter activity. Moreover, the fact that a eukaryotic terminator was present downstream of the target gene enables the expression to be driven by the yeast RNA Pol II and not by T7RNAP. Contrary to Hobl *et al*. [35], the study fails to use a yeast deficient in T7RNAP as a negative control to demonstrate that the expression is driven exclusively by the bacteriophage enzyme. It is thus tempting to think that the translation occurs because it is the yeast RNA pol II transcribing and we believe it is not possible to conclude that the construction of a stable T7 expression system in yeast was achieved, without more data. The authors, however, were successful in increasing the luminescence of their reporter gene by using a viral viroporin, which supports their hypothesis of increasing the membrane permeabilization for enhancing protein production.

Therefore, the challenge for synthetic biology to create a T7RNAP-based expression in yeast remains open; can we direct the yeast protein production from a bacteriophage polymerase?

## 5. Strategies to overcome the need for capping T7RNAP transcripts

### 5.1. Fusion protein T7RNAP and capping enzymes

As discussed previously, mammalian capping enzymes are recruited by the CTD of RNA pol II. Trying to engineer this process, some authors fused the CTD to T7RNAP in an attempt to achieve capping in hosts different from yeast. Surprisingly, none of the studies report protein translation.

Kaneko and coauthors [57] showed that it was possible to create a fusion protein of T7RNAP with the phosphorylated mouse pol II CTD. While a C-terminal fusion rendered the T7RNAP transcription inactive, an N-terminal fusion allowed the catalytic domain to transcribe. They reported the formation of R-loops (nascent transcripts are hybridized into the template DNA during transcription). However, there is no reference in their study to whether the fusion protein was able to generate protein translation. Natalizio and coauthors [58] tested another fusion protein in a similar approach, where they linked the phosphorylated mammalian CTD to T7RNAP. They show that this fusion is not sufficient to achieve the capping of transcripts when compared to the T7RNAP alone, *in vivo*. This suggests that the transcripts will likely not be translated into protein.

These studies suggest the possibility that the CTD is not able to solely recruit the capping enzymes to catalyze the capping of the T7RNAP transcripts [58]. Although the interaction between the CTD of RNA pol II and the capping enzymes is well documented [59–62], previous cryo-electron microscopy (cryo-EM) showed that the CTD is not the only structure interacting with the capping enzymes [61], thus the CTD might not be enough for directing the capping activity to T7RNAP.

In recent years, research focus has increased on mammalian cells, both for gene regulation studies (e.g. for gene therapy) and for eukaryotic protein production. Considering such interest, the creation of a T7RNAP-based expression system in mammalian cells was achieved [12,63]. The system, called C3P3, is based on two components: first, a DNA template with the gene of interest and artificial polyadenylation. Second, a bacteriophage RNA polymerase driving transcription, fused with NP868R, an mRNA capping enzyme from the African swine fever virus (ASFV). This system has proven successful in generating capped and polyadenylated transcripts and achieving protein expression from a T7 promoter.

**Figure 1.**
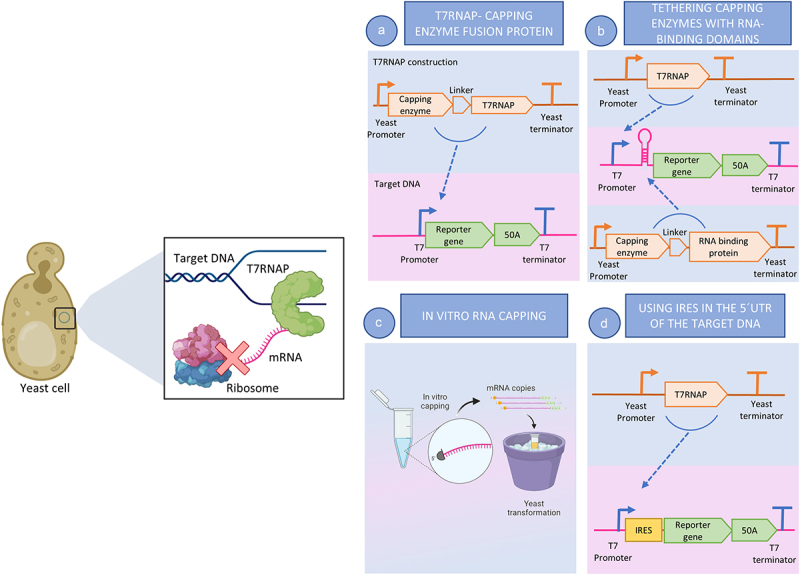
Graphical overview of the different strategies proposed or used in literature to achieve capping of the T7RNAP transcripts. In the graphic on top, nascent RNA is represented in pink (single strand) from T7RNAP, represented in green (as a single subunit enzyme), is not recognized by the eukaryotic ribosome (60S and 40S subunits represented in pink and blue respectively). a) Representation of the genomic construction of a T7RNAP-capping Enzyme fusion protein (blue background) and the target gene (pink background) downstream of the T7 promoter. b) Representation of the genomic construction of an RNA-binding- capping Enzyme fusion protein and the target gene downstream of the T7 promoter and a tethering binding site (TBS). c) Representation of the strategy to achieve *in vitro* capping of the target mRNA and the following yeast transformation with the mRNA. d) Representation of the genomic construction for a T7RNAP and its target gene downstream of the T7 promoter followed by an IRES sequence. Image created in biorender.com.

Although there are differences between mammalian (higher eukaryote) and yeast (lower eukaryote) translation, it is tempting to think that a parallel system in yeast could be the next step to follow and that a similar approach could be utilized for T7RNAP transcripts to be recognized by the yeast ribosomes for protein production (Figure 1a).

### 5.2. Tethering of capping enzymes with RNA binding proteins

Dower and Rosbash [36] proved by a-cap antibody binding assay that the T7 transcripts are not capped when T7RNAP drives the transcription. They hypothesize this to be the reason for the lack of translation in their study. To improve the system, they engineered a 5´stem loop in the transcript, and by expressing a fusion protein (a cap-binding protein with an RNA-binding protein), they could achieve better splicing efficiency. However, the capping status was not reported, therefore it is unknown if their efforts were effective.

This strategy was later adopted in mammalian cells to achieve protein expression by Jäis *et al*. [12] for the creation of a tethering system by fusing viral capping enzymes to RNA binding domains to efficiently create a capping mechanism that allows the translation of T7RNAP transcripts (Figure 1b). It is unclear if this mechanism would also work in yeast.

### 5.3. In vitro capping of T7RNAP transcripts

Another strategy used to cap T7RNAP transcripts is *in vitro* capping. This technology is applied to produce mRNA for therapeutic purposes, which recently resulted in the lightspeed development of mRNA-based vaccines against SARS-CoV-2. *In vitro* capping by CleanCap technology [64] was used for one of the developed vaccines [65]. This technology is based on the initiation of transcription by T7RNAP after a T7 promoter, aided by 5’-cap-containing oligonucleotide primers that resemble the desired cap structure and are complementary to the template DNA sequence at the transcription initiation site. For further information on different *in vitro* cap syntheses refer to the review [66].

Russell, Hambidge and Kirkegarrd [67] used *S. cerevisiae* spheroplasts to introduce mRNA for transient expression of luciferase. They used capped and non-capped transcripts generated by T7RNAP to induce expression and found that the non-capped transcripts were able to generate luminescence, albeit around 10 times lower than the capped ones. Figure 1c represents a possible strategy for generating capped mRNA *in vitro*, followed by a yeast transformation with the capped mRNA. The study also suggests the need for high quantities of RNA for protein production [67], which *in vivo* might be dependent not only on the production but also on the degradation rate.

### 5.4. Use of internal ribosome entry sites

Using IRES to achieve translation without the need for capping, was a strategy used by [35,37], who included an IRES in the 5´UTR of the target gene, downstream of the T7 promoter. While this approach (Figure 1d) is promising since it might allow for bypassing the need for capping, strict controls must be provided, and the results must be carefully interpreted.

Overall, it seems reasonable to think that the capping is at least an enhancer, if not a necessary element, for the creation of a stable expression system in yeast. Thus, a plausible strategy could be a fusion protein comprised T7RNAP and capping enzymes that would be able to cap the transcripts and solve the first step toward protein translation in yeast.

## 6. Strategies to poly-adenylate T7RNAP transcripts

In eukaryotes, the fate of the transcript depends not only on the cap but also on the polyadenylation of the mRNA tail; the 3´-end formation of mRNA seems to influence nuclear export [68,69] and de-adenylation plays an important role in mRNA degradation [70]. Therefore, the proper 3´-end formation should be accounted for when designing the T7RNAP-expression system.

Dower and Rosbash [36] studied the 3´end formation of T7RNAP-generated transcripts in yeast. When the transcripts are terminated by a T7 terminator, they are not polyadenylated and remain nuclear. However, when they tested the transcripts terminated by a polyadenylation signal, they found that transcripts are exported to the cytoplasm and are polyadenylated. This suggests that 3´-end processing might be necessary for nuclear export. This is supported by other studies that correlate proper 3´end formation with nuclear export [71,72].

**Figure 2.**
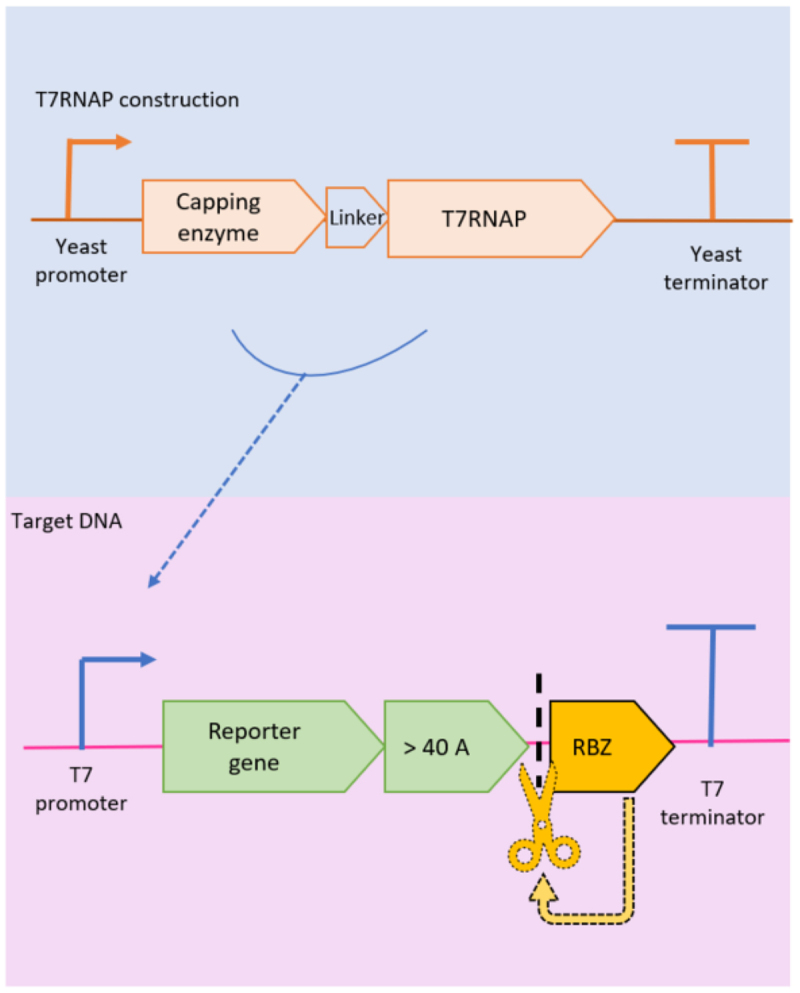
Graphical overview of the genomic construction of a T7RNAP-capping enzyme fusion protein (blue background) and the target gene (pink background) downstream of the T7 promoter. The reporter gene is followed by an artificial poly(a) tail of at least 40 adenosines and a self-cleaving ribozyme (RBZ).

The same group [73] later demonstrated that self-cleaving hammerhead ribozymes in the 3´end of RNA pol II transcripts resulted in the accumulation of nuclear mRNAs, even if some transcripts were able to reach the cytoplasm. They added a synthetic tail of 48 adenosines upstream of the ribozyme which prevented the nuclear accumulation of mRNAs (Figure 2). This demonstrates that nuclear export can be independent of cleavage and polyadenylation, potentially through Pab1-mediated transport [73]. A similar strategy was used by Jäis *et al*. [12], where a 40-adenosine track was followed by a self-cleaving ribozyme (Figure 2) to achieve the artificial polyadenylation of the reporter gene in a T7RNAP-based mammalian expression system.

The cap and the poly(A) tail act synergistically *in vivo* to promote translation, but their individual function is rather inefficient [74]. This inefficiency is notable, especially in the presence of other mRNAs with 5´cap and 3´poly(A) tail competing for translation [75] which would be the case in any context where the yeast is growing while producing the intended protein.

These findings suggest that a synthetic poly(A) tail upstream of a ribozyme element in the DNA template might suffice for the nuclear export of the transcripts. Even if the association with nuclear export is not clear, and some of the transcripts might reach the cytoplasm, the interactions between the poly(A) tail of the transcripts and Pab1 in translation, strongly suggest the need for a synthetic poly(A) tail, especially if the target translation will be via ‘closed-loop model’ (cap-dependent).

## 7. Expression system design: nuclear or cytoplasmic location?

It has been demonstrated that an NLS incorporated in the N-terminal of the protein directs the T7RNAP into the yeast cytoplasm both in *S. cerevisiae* and in *P. pastoris* [34,35]. However, it should be noted that there might be advantages and disadvantages in placing the target gene in the nuclear chromosome instead of in a plasmid.

The chromosomal integration of the target gene downstream of the T7 promoter offers a stable and controllable system [76]; however, chromatin accessibility in the nucleus could interfere with T7RNAP efficiency [28,33].

The nuclear export of the transcripts to the cytoplasm might be another limiting step in the protein expression process. Processes of quality control and degradation assure that mRNA exported to the cytoplasm is capped, polyadenylated, and spliced. Several mechanisms prevent aberrant mRNA from reaching the cytoplasm, being discarded during the early steps of their biogenesis [68]. In fact, Yan *et al*. [37] demonstrate that an increase in protein production is possible by increasing the nuclear membrane permeabilization in *S. cerevisiae*.

Following the genomic location, the promoter of choice to express the T7RNAP will also differ depending on whether the pathway must be active during cell growth (constitutive promoter) or if a certain degree of control is needed for the expression (inducible promoter). The integration of the T7RNAP in the yeast genome, and the introduction of the target gene in a vector (analogous to the pET expression system in *E. coli* [9]) could allow for a more efficient expression system since the nuclear export of the transcript would not have to be accounted for. In contrast, using a vector for expression could mean a less stable system and more variability in protein expression levels, depending on the plasmid copy number [76].

The choice of yeast for the design of the protein expression system should also be considered. To date, *S. cerevisiae* and *P. pastoris* have been used for the design of such a system. While *S. cerevisiae* offers the advantage of being a well-studied organism, the use of *P. pastoris* as a protein expression system is steadily increasing and is already a key host for the industrial production of biopharmaceuticals [17].

In prokaryotes, the interplay between the T7RNAP and its promoter has been well described [9,77,78]. In yeast, processes such as transcription and replication are regulated by chromatin structure, where the condensed heterochromatin can inhibit gene expression [79]. T7RNAP can access its promoter in *S. cerevisiae* chromatin [33]. In *P. pastoris*, T7RNAP recognized its promoter present in a vector [35], thus it is still unclear if the enzyme can access and transcribe effectively from *P. pastoris* chromatin. Moreover, *P. pastoris* centromere structure differs from *S. cerevisiae* [80]. Previous studies have shown that chromatin structure can affect T7RNAP transcription, which is less effective in nuclear chromatin of higher eukaryotes, contrary to lower eukaryotes [28,55,56]. Thus, considering the interplay between the polymerase and the promoter should also be considered when choosing between the integration of the target gene or the use of a plasmid for the expression.

## 8. Conclusion and future perspectives

The studies highlighted here show the progress made, but also show the unresolved challenges toward a T7RNAP-based expression system in yeast. Nevertheless, we believe that identifying the challenges is a way forward to solving them, and we are thus certain that as with the mammalian T7-based system, a T7-yeast-based expression system will also be established. We expect the future progression of this system to further develop with the exploitation of genome editing tools such as CRISPR/Cas [20,81,82]. Providing the benefits of a host-independent transcription to yeast protein expression, will not only be a useful tool for synthetic biology, but it will also contribute to understanding the genetic regulation in eukaryotic systems and will ultimately increase the versatility of yeast in the sustainable production of different metabolites.
